# Prediction of Perianal Fistula in Crohn’s Disease by Computed Tomography Enterography

**DOI:** 10.5152/tjg.2024.22850

**Published:** 2024-03-01

**Authors:** Genghuan Ni, Hongwei Zhao, Haiyan Shen, Senjuan Li, Weiqun Ao

**Affiliations:** 1 Department of Radiology, The Second Affiliated Hospital of Jiaxing University, Jiaxing, Zhejiang Province, China; 2 Department of Inflammatory Bowel Disease Center, The Second Affiliated Hospital of Jiaxing University, Jiaxing, Zhejiang Province, China; 3 Department of Anorectal Surgery, The Second Affiliated Hospital of Jiaxing University, Jiaxing, Zhejiang Province, China; 4 Department of Radiology, Tongde Hospital of Zhejiang Province, Hangzhou, Zhejiang Province, China

**Keywords:** Inflammatory bowel disease, Crohn’s disease, perianal fistula, computed tomography enterography

## Abstract

**Background/Aims:**

The purpose of this study was to investigate whether computed tomography enterography can be used to predict the presence of perianal fistula in Crohn’s disease patients.

**Materials and Methods:**

According to the presentation of perianal fistula or not, this study divided retrospectively included Crohn’s disease patients into 2 groups. The disease duration, incidence of involved intestinal segments, and scoring of the activity of the lesions in all patients were statistically analyzed to explore significant factors between the 2 groups. The statistically significant findings identified in the univariate analysis were incorporated into the multivariate analysis. Logistic regression models were subsequently constructed to assess the predictive factors associated with the occurrence of perianal fistula in individuals with Crohn’s disease.The contribution of each factor to the outcome variable was confirmed by the nomogram. The clinical utility of the nomogram was confirmed by calibration and decision curves.

**Results:**

There were 40 cases with perianal Crohn’s disease and 58 without perianal Crohn’s disease. After univariate and multivariate analysis, disease duration (early stage of Crohn’s disease), ascending colon, and rectum were identified as the independent predictive factors for perianal fistula in Crohn’s disease patients. The clinical utility of the nomogram was effective, which implied potential benefits for Crohn’s disease patients.

**Conclusion:**

Computed tomography enterography can be used to predict the presence of perianal fistula in Crohn’s disease patients by analyzing the location and the stage of the disease.

**Main Points**

- Growing emphasis has been put on Perianal Crohn’s Disease (PCD). We conducted a case control study of PCD and N-PCD patients.
- We explored the factors associated with the presence of perianal fistula (PF) by computed tomography enterography (CTE) and found that PF is more likely to occur in the early stage of Crohn’s Disease (CD) and the development of PF is closely related to the affected intestinal tract.
- CTE supports the detection and diagnosis of PCD. So we systematically summarized the CTE characteristics of PCD patients to understand the disease comprehensively. We propose that disease process combined with the affected intestinal site of CD contribute to the detection of PF in CD patients, therefore provides evidence for further treatment of PCD patients.

## Introduction

Crohn’s disease (CD) is a type of inflammatory bowel disease with a specific clinical course.^1,2^ With the acceleration of industrialization and urbanization, as well as changes in the environment and diet structure, the incidence rate of CD, once considered to be a high incidence in Western countries, has shown unprecedented growth in China.^3,4^ Perianal CD (PCD) is a form of CD involvement that shows poor prognosis.^5^ Perianal Crohn’s disease patients have a wider extent of lesions, more complex lesion behaviors,^6^ longer hospital stays, increased perianal and abdominal surgery rates,^7^ worse postoperative outcomes, and significantly reduced quality of life.^8^ There is evidence that the prevalence of PCD in Asia is higher than in Western countries, and the progression of PCD may be different between patients from the East and the West.^9,10^ More attention should be paid to PCD patients in these new-onset CD populations because of the growing trend of CD in China in the past few decades. Clinicians may miss the diagnosis in PCD patients only based on their medical history and symptoms. Therefore, further studies need to be conducted on PCD patients or patients who are likely to develop PCD.

The study of PCD through pelvic MRI has found that a highly complex perianal fistula (PF) is a characteristic of PCD.^11^ Complex PF is one of the most common manifestations of PCD and is considered a poor prognostic factor in the progression of CD.^12,13^ Magnetic resonance enterography is an accurate and nonradiation examination for the simple evaluation of PF and CD. However, CD patients are usually admitted with abdominal pain. Because of the fast imaging speed, wide range, and few contraindications of computed tomography enterography (CTE), the first abdominal imaging examination is mostly CTE.^14^ Computed tomography enterography has become a widely accepted method for a detailed evaluation of the small bowel of CD patients.^15^ Due to its relatively poor soft tissue resolution, CTE examination of the pelvic floor might fail to detect the presence of PF. Meanwhile, the risk factors for PCD have not been fully studied. It is generally believed that there is an association between colorectal lesions and PF,^16^ but there is no clear conclusion on the correlation between other intestinal lesions and PF. It is generally believed that PCD patients will have significantly more intestinal inflammatory activity than other CD patients,^7^ but it is still controversial whether PF is related to intra-abdominal penetrating lesions (fistulas and abscesses). The age of onset plays an important role in the course of CD and is associated with the incidence of PF,^10^ but whether the incidence rate of PF is the same at different stages of CD has not been reported. Therefore, studying the CTE manifestations of PCD patients and the risk factors for PCD is helpful for further research on PF’s existence in CD patients or CD patients who may develop PF.

In this study, we retrospect clinical data and CTE in patients, explore the risk of PF incidence and investigate the role of CTE in predicting PF existence in CD patients, aiming to provide a reference for the clinical diagnosis, treatment, and evaluation of PCD.

## Materials and Methods

### Patient Selection

This study was conducted in accordance with the Declaration of Helsinki in 1964 and approved by the Institutional Ethics Committee of The Second Affiliated Hospital of Jiaxing University (approval number JXEY-2020JX092). Informed consent was waived for this retrospective study.

**Figure 1.**
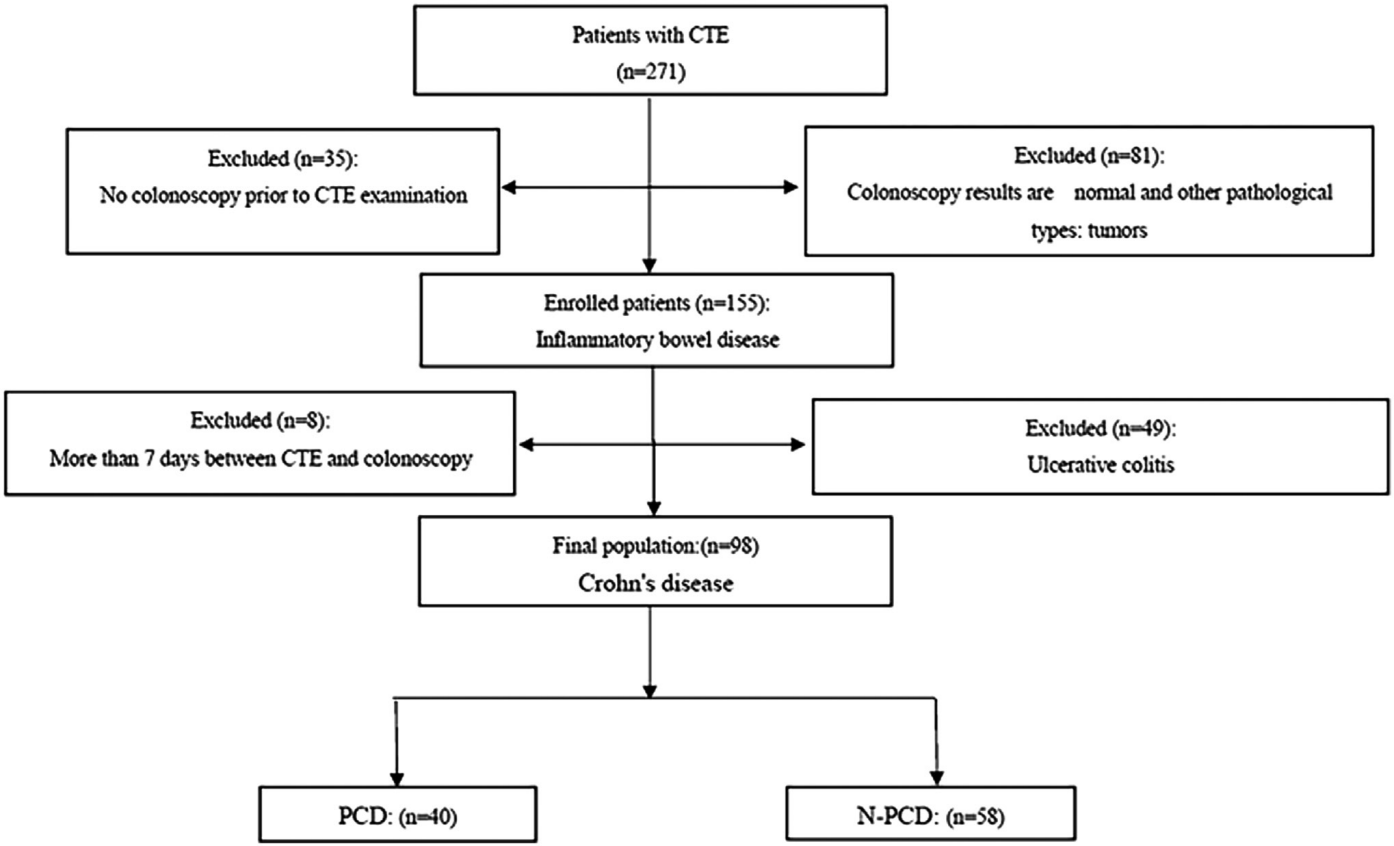
Flow diagram of enrolled patients.

This study retrospectively analyzed CD patients who met the following criteria from July 2017 to March 2021 in The Second Affiliated Hospital of Jiaxing University. The inclusion criteria were as follows: (i) the diagnosis of CD was confirmed by colonoscopy and pathology; (ii) PF was the most common manifestation of PCD, and it was often represented by PF, in this study, only PCD patients with PF were included, the diagnosis of PF was confirmed by clinical examination and MRI; (iii) the data of CTE examination were complete; (iv) the interval between CTE and colonoscopy was no more than 7 days; and (v) the interval between CTE and diagnosis of PF was no more than 30 days. The exclusion criteria were as follows: (i) non-CD patients, (ii) nonanal fistula patients with the perianal disease, and (iii) unclear colonoscopic or pathologic results. According to the presence or absence of PF at the time of CTE examination, the enrolled patients were assigned into 2 groups: a PCD group and a nonperianal CD (N-PCD) group (Figure 1).

### Image Acquisition

All patients underwent full abdominal CTE scan from the diaphragm dome to the level of the lower edge of the pubic symphysis. The patients were not allowed to eat for more than 12 hours and received bowel cleansing treatment before CTE. Within 1 hour before CTE, each patient took 1200 mL of 2.5% isotonic mannitol solution orally in 3 doses at 15-minute intervals. The patient was placed in the supine position and held their breath during the scanning. The scanning was performed with a SOMATOM Definition Flash CT scanner (Siemens Healthineers, Forchheim, Germany) at the following parameter settings: tube voltage = 120 kV, tube current in automatic mode, pitch = 1.0, slice thickness = 8 mm, interslice gap = 8 mm, reconstructed image slice thickness = 2 mm, and convolution value = B21f. For contrast-enhanced scanning, a nonionic contrast agent (Ioversol, 350 mg/mL) was injected through the median cubital vein with a high-pressure syringe (dose = 1.5 mL/kg; injection flow rate = 2.5-3.0 mL/s). The arterial-phase scan was performed 23-25 seconds after the injection of the contrast agent, and the venous-phase scan was performed 60-70 seconds after the injection of the contrast agent.

### Image Analysis

Two radiologists with more than 10 years of diagnostic experience in abdominal imaging blindly assessed the locations of lesions in the intra-abdominal bowel segment and the comprehensive CTE scores in the PACS system. The 2 negotiate and reach a consensus as the final result.

The digestive tract was divided into 9 intestinal segments, i.e., the jejunum, proximal ileum, mid-distal ileum, ileocecal junction, ascending colon, transverse colon, descending colon, sigmoid colon, and rectum. The lesion locations of all patients were recorded and classified according to the Montreal classification. A 4-point scale was used to evaluate imaging indicators such as the degree of mesenteric fat exudation, comb sign, mesenteric lymph node enlargement, intestinal wall enhancement, and degree of intestinal stricture^17^ (see Table 1 for details), from which the comprehensive CTE score was calculated. The abovementioned imaging indicators were defined as follows:^18^ (i) peri-intestinal fat exudation: increased mesenteric CT density and blurred edges; (ii) comb sign: increased, thickened, and tortuous small blood vessels supplying the intestinal wall; (iii) mesenteric lymph node enlargement: mostly long, ovoid lymph nodes with the short-axial diameter exceeding 10 mm; (iv) intestinal wall enhancement: the attenuation value of the intestinal wall in the lesioned intestinal segment was greater than that of the adjacent normal intestinal segment by more than 10 HU on the contrast-enhanced CTE image; and (v) intestinal wall thickening: the measured intestinal wall thickness was greater than 3 mm in an intestinal segment that was fully dilated by the fluid. The thickening and enhancement of the intestinal wall with the most severe lesions were recorded. (vi) The luminal stricture was defined as a 50% reduction in the diameter of the lumen compared with that of the normal adjacent intestinal loop and evident dilation of the upstream intestinal segment (i.e., ≥3 cm). (vii) The typical feature of penetrating diseases is the formation of a fistula (an abnormal connection between 2 epithelial surfaces). (viii) Anal fistula was defined as a fistula formed by the communication between the anorectal canal and the perianal skin.

### Statistical Analysis

Statistical analysis was performed with Statistical Package for the Social Sciences 22.0 software (IBM Corp.; Armonk, NY, USA). Continuous variables confirmed to follow a normal distribution by the Kolmogorov–Smirnov test was expressed as the mean and standard deviation and was compared between groups using the Student’s *t*-test. Nonnormally distributed continuous variables are expressed as the median with quartiles and were compared between groups using the rank sum test. Count data were compared between groups using the cross-tab chi-squared test or Fisher’s exact test. The logistic regression model was used to evaluate the predictors of response. The statistically significant results in the univariate analysis were input into the multivariate analysis. R software was used to draw nomograms, and the R language rms software package and rmda software package were used for calibration curve analysis and decision curve analysis (DCA), respectively. Interobserver reproducibility of CTE was assessed by using intraclass correlation coefficient analysis. A *P*-value <.05 was considered statistically significant.

## Results

### Patient Characteristics

A total of 98 patients who met the study criteria in the medical records from July 2017 to March 2021 were included in the study. The clinical symptoms mainly included abdominal pain, diarrhea, bloody stools, fever, anorexia, perianal pain, and fluid discharge. The PCD group included 40 patients (35 males and 5 females), with a mean age of 30.1 ± 11.8 years, an onset age of 30.2 ± 11.5 years, and a median disease duration of 1.5 (1.0, 12.0) months. The N-PCD group included 58 patients (41 males and 17 females), with a mean age of 35.0 ± 10.8 years, an onset age of 34.0 ± 11.0 years, and a median disease duration of 12 (1.0, 17.3) months. The 2 groups were not significantly different in age, age of onset, sex composition, or C-reactive protein (CRP) (*P* > .05), but were significantly different in disease duration (*P* = .011) and the proportion of patients with a disease duration <6 months (*P* = .040; odds ratio, OR: 2.361; 95% confidence interval, CI: 1.003-5.395) (see Table 2 for details).

### Results of Distribution of Affected Sites

**Figure 2.**
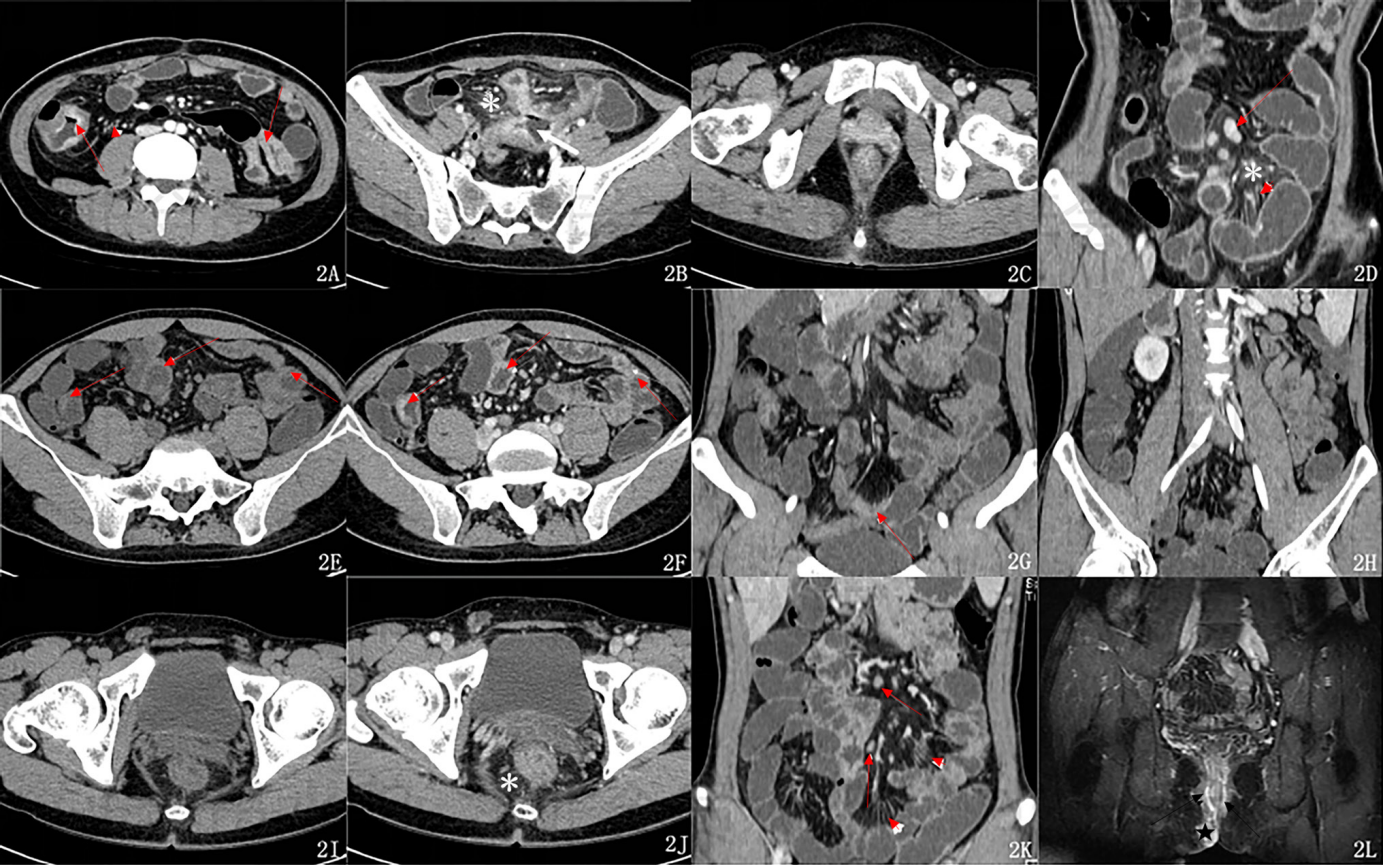
Computed tomography enterography imaging features of the two groups.

Ileocecal involvement accounted for the highest proportion in both groups, but the 2 groups were not significantly different in ileocecal involvement (*P* >.05). The incidence rates of proximal ileal and rectal involvement were significantly higher in the PCD group than in the N-PCD group (*P* = .027, *P* = .004). The incidence rate of ascending colon involvement was significantly lower in the PCD group (*P* = .025). The 2 groups were not significantly different in the involvement of other sites (Figure 2A–L) or the Montreal classification (see Table 2 for details).

### Computed Tomography Enterography Gastrointestinal Lesion Activity Comprehensive Score Results

The mean comprehensive CTE score of the PCD group (9.40 ± 2.58) was significantly higher than that of the N-PCD group (8.03 ± 2.98). However, if PF is not considered, the 2 groups were not significantly different in the comprehensive CTE score or the mean score of any individual item (see Table 3 for details) (Figure 2A–L).

### Predictors of Perianal Fistula

Logistic regression analysis was performed to determine the independent predictors of PF. Univariate analysis showed that disease duration, ascending colon involvement, rectal involvement, and proximal ileal involvement were significant predictors for PF in CD patients. Only significant variables in univariate analysis were used in multivariate modeling. Multivariate analysis based on age and sex showed that disease duration, rectal involvement, and ascending colon no-involvement had significant value in predicting the development of PF in CD patients. However, the proximal ileal involvement, a significant variable in the univariate analysis, was not significant in the multivariate analysis (Table 4). A binary unconditional logistic regression model was used to investigate the influencing factors of the presence of PF in CD patients.

### Clinical Application of Computed Tomography Enterography

**Figure 3.**
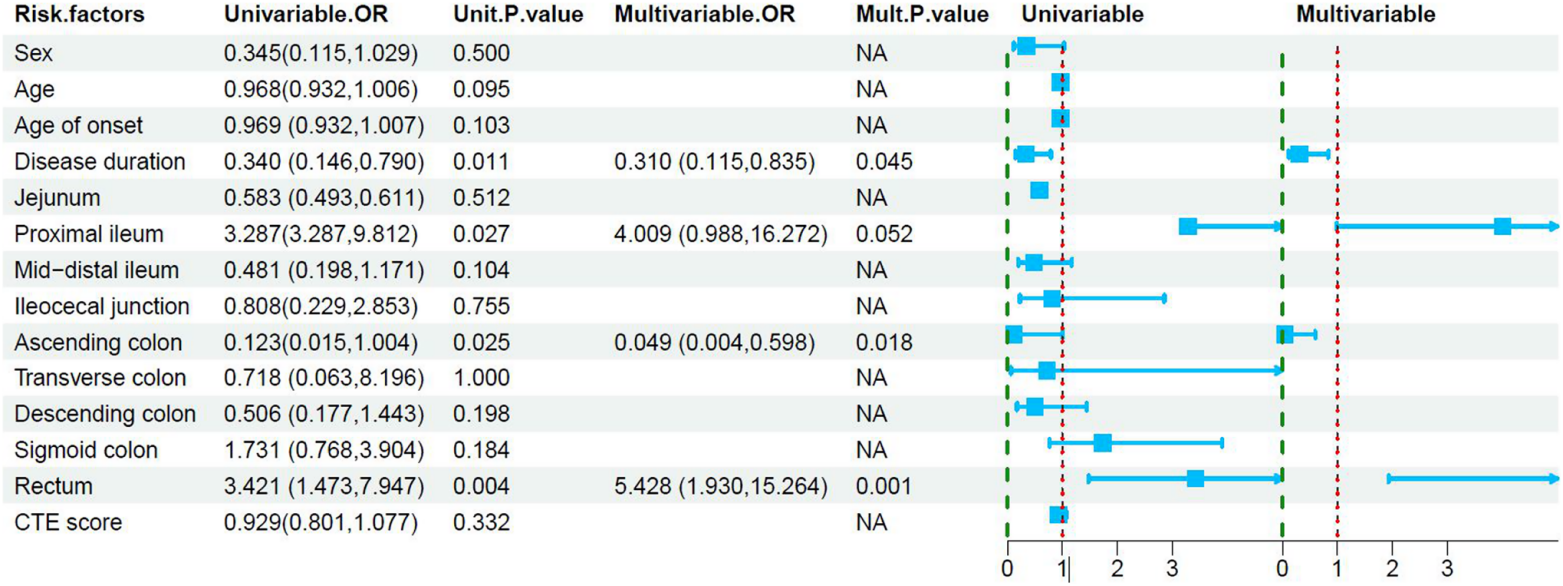
Results of univariate and multivariate logistic regression analyses of significant predictors for perianal fistula in Crohn’s disease patients.

**Figure 4.**
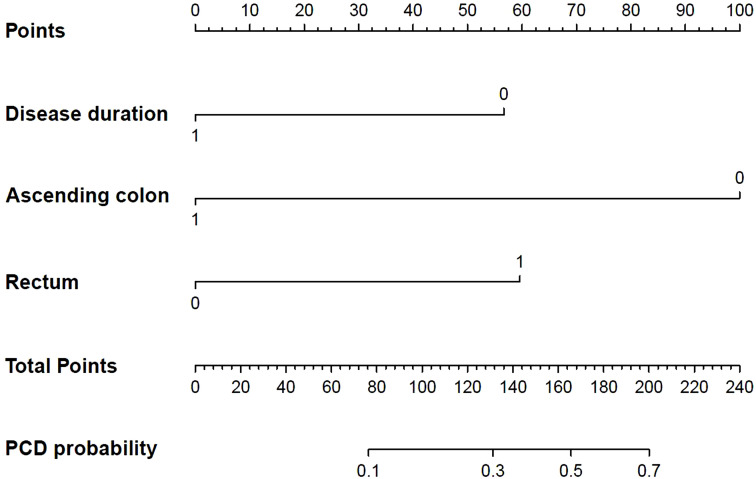
The nomogram of computed tomography enterography predicting perianal Crohn’s disease.

**Figure 5.**
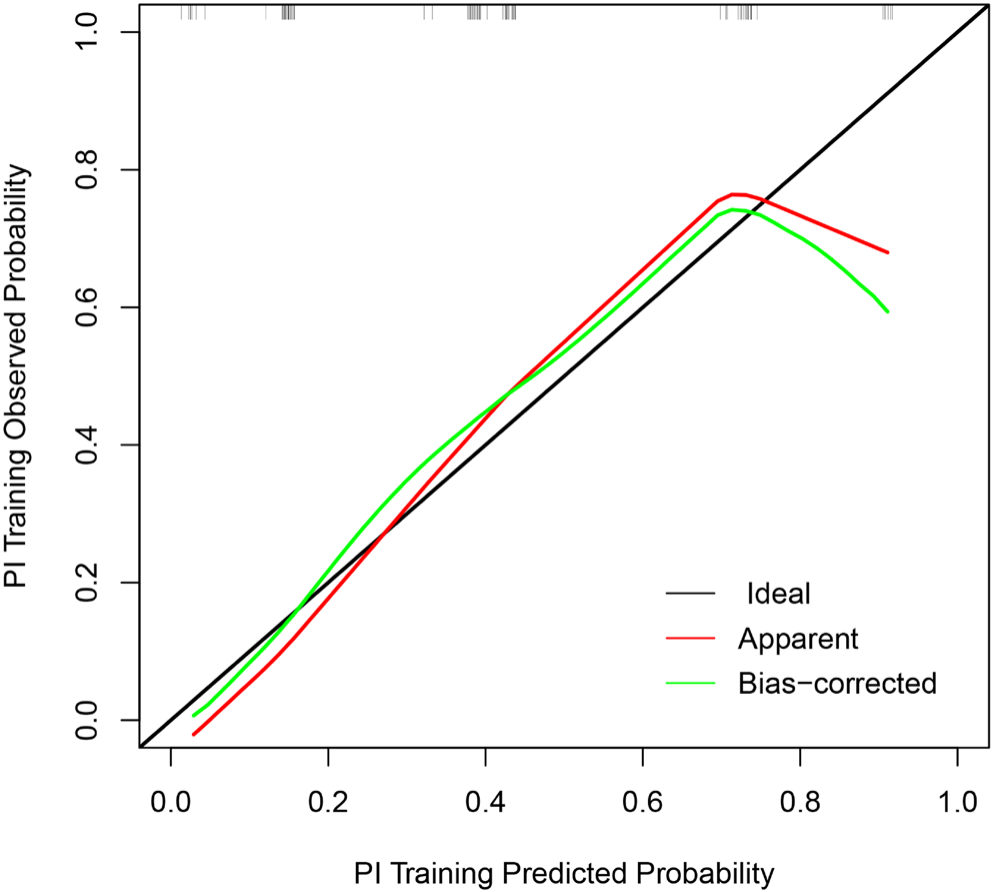
The calibration curve of the prediction model.

A nomogram was constructed from the 4 selected independent predictors to reflect the contribution of each factor to the outcome variable (Figure 3). Meanwhile, the Hosmer–Lemeshow goodness-of-fit test showed that the model had good reliability (*P* = .342). The prediction model calibration curve showed that the slope of the fitting curve of the prediction model was close to 45°, which was parallel to the Idea line, and the model performance was good (Figure 4). The DCA decision analysis curve showed that, under certain conditions, using the CTE to predict PF in CD patients was more beneficial than identifying all CD patients with PF or without PF (Figure 5). Interobserver reproducibility assessment showed high consistency of the CTE measurements between the 2 observers without significant differences in the mean (see Table 5 for details).

## Discussion

Crohn’s disease is a lifelong disease caused by the interaction between genetic and environmental factors, and the exact etiology is still unclear.^18^ Approximately one-quarter of CD patients may develop perianal disease.^13^ The presence of perianal disease in CD patients usually indicates a higher hospitalization rate, more invasive abdominal intestinal lesions, and a higher risk of abdominal surgery.^1,13,19^

Our data showed that both PCD patients and N-PCD patients had the characteristics of CD, with the ileocecal junction as the most commonly affected site.^20^ However, we found that the rectal involvement rate in the PCD group was significantly higher than that in the N-PCD group. In the presence of rectal involvement, the OR of PF in CD patients was significantly higher. This is consistent with the previous results^21^ that up to 92% of CD patients with rectal involvement developed PF.^22^ The large and complex microbiota in PCD fistulas leads to a high incidence rate of rectal involvement.^23^ In addition, CD-induced active proctitis has a negative impact on perianal diseases,^24^ and the 2 interact with each other, resulting in high incidence rates of proctitis and PF. Therefore, CD patients with rectal involvement should be considered at high risk of PF. The presence of proctitis should be confirmed immediately after the diagnosis of CD, as this information is critical to predicting the risk of PF in CD patients.

There is much controversy about the relationship between PCD and the proximal small intestine.^25,26^ Perianal Crohn’s disease is least common in patients with isolated ileal disease (12%) or ileocolonic disease (15%) and is uncommon in adult patients with small bowel or ileal CD.^23^ In our samples, the incidence rate of proximal ileal involvement in the PCD group (27.50%) was significantly higher than that in the N-PCD group (10.35%). Although the proximal small bowel (jejunum + proximal ileum) involvement rate did not reach a significant difference between the 2 groups, it was higher in the PCD group than in the N-PCD group. The proximal ileal involvement rate in PCD patients showed a high OR in univariate analysis but was not significant in multivariate analysis. The difference in CRP did not reach a significant difference between the 2 groups. Our results only support part of the results of Xavier et al,^13^ suggesting that further studies are needed to explain the relationship between different affected sites and PCD.

The probability of developing PF is 5%-21% in adults with abdominal CD and 8%-27% in children with abdominal CD.^26,27^ The study of Maccioni et al^16^ also showed that children with CD had a higher risk of developing rectal CD and a higher prevalence of perianal diseases than adults with CD. These findings indicate that the age of onset plays an important role in the development of perianal disease in CD. Our data showed that the age of onset was slightly but not significantly different between the 2 groups, but the disease duration in the PCD group was significantly shorter than that in the N-PCD group. The patients with disease duration below 6 months accounted for 62.5% of the PCD group and only 41.38% of the N-PCD group. Therefore, we can draw the following conclusions: The incidence rate of PF is also different in different stages of CD, the prevalence being higher in the early stage of CD than in the later stages. In 1 study, 17.2% of patients developed perianal lesions within 6 months before the diagnosis of CD; 26.9% of patients developed perianal diseases from 6 months before the diagnosis of CD to 6 months after the diagnosis.^28^ This explains the short disease duration in the PCD group. Perianal fistula is prone to occur in the early stage of CD, so it is an early sign of CD. Patients diagnosed with PF are often at the early stage of CD when they undergo a full abdominal examination due to PF to screen for abdominal intestinal diseases. At the same time, our study showed that the incidence rate of ascending colon involvement in the PCD group (2.5%) was significantly lower than that in the N-PCD group (17.24%), and the OR of PCD in patients with ascending colon involvement was lower. We propose that because the onset of PCD patients was in the early stage of CD, the extent of intestinal involvement was relatively small, and the probability of ascending colon involvement was relatively low. From our findings, we can conclude that the stage of disease duration is a significant predictor of the probability of developing PF in CD patients, which of course needs to be further verified by more samples.

Adler et al^28^ and Xavier et al^13^ showed that patients with perianal diseases might have a greater burden of inflammation and more complex behaviors, even in the gastrointestinal segments usually unrelated to CD. Zhao et al^1^ found that PCD was correlated with the development of an internal fistula, but other studies showed that PF was more common in patients with no internal fistula or stricture.^26^ Our results showed that the development of PF in CD patients was not correlated with the behavioral manifestations of CD. The 2 groups were not significantly different in the CTE scores of the rating items other than a fistula. If PF was not considered, the comprehensive CTE score was not significantly different between the 2 groups. According to the Montreal classification (behavior), the majority of PCD patients in our study had nonadhesive and nonpenetrating diseases (B1). Therefore, further studies are needed to investigate the different mechanisms that induce the development of PF and internal fistula in different individuals.

Computed tomography enterography can be used to quickly and comprehensively examine CD patients or suspected CD and thus has become one of the most effective methods for small bowel imaging in CD patients and a supplement to endoscopy. It is useful in the clinical diagnosis of CD, the determination of the affected sites, and the screening of complications.^15^ This study showed that the early stage of CD and rectal involvement were high-risk factors for the development of PF in CD patients. Based on the predicted probability and the actual occurrence of PF in CD patients, the logistic regression fitting model was made. The model performed excellently at predicting the risk of PF in CD patients. Therefore, CTE can guide the clinical prediction of the development of PF in CD patients based on disease duration. Because of the soft tissue resolution of CTE, it was difficult to detect PF in the scan of the pelvic floor and perineum, and it was possible to ignore perianal lesions clinically. Therefore, under the premise of combining the duration of the disease, the use of this model could help CTE to improve the detection rate of PCD patients, thereby helping to provide patients with better treatment and benefiting PCD patients. The DCA curve has also verified this.

Our study has a few limitations. First, because it was a single-center study, the number of included cases was moderate. Multicenter validation studies are needed. Second, this study was only a retrospective study and did not conduct a prospective study of the risk factors and probability of PF. In this study, 5 patients with rectal involvement in the N-PCD group also developed PF during subsequent follow-up, but due to the small sample size, they were not used as the test group, which needs more cases for validation. Finally, our assessment did not include the effects of different treatments on disease progression and evolution.

Our results indicate that the development of PF in CD patients is closely related to the affected intestinal sites, but is relatively unrelated to disease behavior and that PF is prone to occur in the early stage of CD. When combined with clinical data and the involved sites in CD patients, CTE was helpful in the detection of PF in CD patients, which could provide comprehensive information for further treatment of PCD patients.

## Data Availability:

All data generated or analyzed during this study are included in this article.

## Figures and Tables

**Table 1.** Comprehensive Scoring Criteria for Computed Tomography Enterography

Scoring Item	0	1	2	3
Mesenteric fat exudation	No	Mild turbidity at 1 site	Evident turbidity at 1 site	Evident turbidity at multiple sites
Comb sign	No	Minimum range	Segmental dilation with mild tortuosity	Diffuse dilation with severe tortuosity
Lymph node enlargement	No	Regional lymphadenopathy	Distant lymph node enlargement	Both
Fistula	No	Ileocecal fistula	Perianal fistula	Both
Intestinal wall enhancement	No	Mild	Moderate	Significant
	0-10 HU	10-20 HU	21-40 HU	>40 HU
Intestinal wall thickness	<3 mm	3-5 mm	5-9 mm	>9 mm
Intestinal stricture	No	Low-grade partial obstruction	Severe partial obstruction	Complete obstruction

CTE, computed tomography enterography; HU, Hounsfield unit.

**Table 2.** Statistical Results of Sex, Age, Age of Onset, Onset Location, and Montreal Class of CD Patients in the 2 Groups

Item	PCD (n = 40)	N-PCD (n = 58)	*P*
Sex			
				Male	35 (87.50% )	41 (70.69%)	.050^c^3
Female	5 (12.50% )	17 (29.31%)					
Age (years)	30.1 ± 11.8	35.0 ± 10.8	.095^b^3
Age of onset (years)	30.2 ± 11.5	34.0 ± 11.0	.103^b^3
Disease duration (months)	1.5 (1, 12)	12.0 (1, 17.3)	.011^a^3
CRP (mg/L)	11.78 (2.20, 19.06)	5.17 (0.86, 25.46)	.520^c^3
Disease duration			
				<6 months	25 (62.50% )	24 (41.38%)	.040^c^3
>6 months	15 (37.50%)	34 (58.62%)					
Involved site			
				Jejunum	0 (0% )	2 (3.45% )	.051^c^3
				Proximal ileum	11 (27.50%)	6 (10.35% )	.027^c^3
				Mid-distal ileum	25 (62.50%)	45 (77.59%)	.104^c^3
				Ileocecal junction	35 (87.50%)	52 (89.66%)	.740^c^3
				Ascending colon	1 (2.5%)	10 (17.24%)	.025^c^3
				Transverse colon	1 (2.5% )	2 (3.45%)	1.000^c^3
				Descending colon	6 (15.00%)	15 (25.86%)	.198^c^3
				Sigmoid colon	22 (55.00%)	24 (41.38%)	.184^c^3
Rectum	25 (62.50% )	19 (47.50%)	.004^c^3
Montreal classification (location)			
				L1	17 (42.50% )	33 (56.90%)	.317^c^3
				L2	3 (7.50% )	2 (3.45%)	
L3	20 (50.00% )	23 (39.65%)					

CRP, C-reactive protein; N-PCD, nonperianal Crohn’s disease; PCD, perianal Crohn’s disease.

^a^Wilcoxon rank-sum test.

^b^Student’s *t*-test.

^c^Cross-tab chi-squared test.

**Table 3.** Results of Comprehensive Computed Tomography Enterography Score and Montreal Class (Behavior) of the 2 Groups of Crohn’s Disease Patients

Item	PCD	N-PCD	*P*
Mesenteric fat infiltration	0 (0, 1)	0 (0, 1)	.297^a^3
Comb sign	1 (1, 2)	1 (1, 2)	.128^a^3
Lymph nodes	2 (1, 2)	1 (1, 2.75)	.849^a^3
Fistula (including PF)	2 (2, 2)	0 (0, 0)	<.001^a^3
Fistula (excluding PF)	0 (0, 0)	0 (0, 0)	.584^a^3
Degree of enhancement	2 (2, 3)	2 (1, 3)	.175^a^3
Intestinal wall thickness	2 (1, 3)	2 (1, 3)	.963^a^3
Stricturing	0 (0, 0)	0 (0, 1)	.348^a^3
Total comprehensive CTE score (including PF)	9.40 ± 2.58	8.03 ± 2.89	.021^b^3
Total comprehensive CTE score (excluding PF)	7.48 ± 2.49	8.03 ± 2.89	.332^b^3
Montreal classification (behavior)			
				B1	34 (85.00%)	45 (77.59%)	.750^c^3
				B2	3 (7.50%)	10 (17.24%)	
B3	3 (7.50%)	3 (5.17%)					

CTE, computed tomography enterography; N-PCD, nonperianal Crohn’s disease; PCD, perianal Crohn’s disease; PF, perianal fistula.

^a^Wilcoxon rank-sum test.

^b^Student’s *t*-test.

^c^Cross-tab chi-squared test.

**Table 4.** Results of Univariate and Multivariate Logistic Regression Analyses of Significant Predictors for Perianal Fistula in Crohn’s Disease Patients

Risk Factors	Univariate Analysis			Multivariate Analysis		
	OR	95% CI	*P*	OR	95% CI	*P*
Sex	0.345	0.115-1.029	.500	3	3	3
Age	0.968	0.932-1.006	.095	3	3	3
Age of onset	0.969	0.932-1.007	.103	3	3	3
Disease duration	0.340	0.146-0.790	.011	0.310	0.115-0.835	.045
Jejunum	0.583	0.493-0.611	.512	3	3	3
Proximal ileum	3.287	3.287-9.812	.027	4.009	0.988-16.272	.052
Mid-distal ileum	0.481	0.198-1.171	.104	3	3	3
Ileocecal junction	0.808	0.229-2.853	.755	3	3	3
Ascending colon	0.123	0.015-1.004	.025	0.049	0.004-0.598	.018
Transverse colon	0.718	0.063-8.196	1.000	3	3	3
Descending colon	0.506	0.177-1.443	.198	3	3	3
Sigmoid colon	1.731	0.768-3.904	.184	3	3	3
Rectum	3.421	1.473-7.947	.004	5.428	1.930-15.264	.001
Total comprehensive CTE score (excluding PF)	0.929	0.801-1.077	.332	3	3	3

CD, Crohn’s disease; CTE, computed tomography enterography; OR, odds ratio; PF, perianal fistula.

**Table 5.** Interobserver Reproducibility Assessment of the CTE Measurements Between the 2 Observers

Item	Observer 1	Observer 2	ICC	*P*
Mesenteric fat infiltration	0 (0, 1)	0 (0, 1)	0.853	<.001
Comb sign	1 (1, 2)	1 (1, 2)	0.843	<.001
Lymph nodes	2 (1, 2)	2 (1, 2)	0.888	<.001
Degree of enhancement	2 (2, 3)	2 (2, 3)	0.822	<.001
Intestinal wall thickness	2 (1, 3)	2 (1, 3)	0.825	<.001
Stricturing	0 (0, 0)	0 (0, 0)	0.855	<.001
Fistula (including PF)	0 (0, 2)	0 (0, 2)	0.997	<.001
Fistula (excluding PF)	0 (0, 0)	0 (0, 0)	0.997	<.001
Total comprehensive CTE score (including PF)	8.69 ± 2.79	8.87 ± 2.85	0.945	<.001
Total comprehensive CTE score (excluding PF)	7.94 ± 2.69	8.11 ± 2.76	0.942	<.001

CTE, computed tomography enterography; PF, perianal fistula.
